# Implementation fidelity of a smoke-free workplace intervention in a private medical company: A mixed-methods process evaluation

**DOI:** 10.18332/tpc/162878

**Published:** 2023-05-26

**Authors:** Sofie K. B. Rasmussen, Lærke P. Lidegaard, Charlotta Pisinger, Nina F. Johnsen, Maria Kristiansen

**Affiliations:** 1 Center for Clinical Research and Prevention, Bispebjerg-Frederiksberg University Hospital, Capital Region of Denmark, Frederiksberg, Denmark; 2 Department of Research, Danish Heart Foundation, Copenhagen, Denmark; 3 Department of Public Health, Faculty of Health and Medical Sciences, University of Copenhagen, Copenhagen, Denmark; 4 Department of Public Health and Center for Healthy Aging, Faculty of Health and Medical Sciences, University of Copenhagen, Copenhagen, Denmark

**Keywords:** smoke-free policies, smoke-free workplace, smoke-free work hours, tobacco-free workplace, implementation research, mixed methods study

## Abstract

**INTRODUCTION:**

Smoke-free workplaces are considered an important part of tobacco control strategies. The purpose of this study was to evaluate implementation fidelity and explore the significance of social and contextual factors for the implementation of a strict smoke-free workplace intervention in a large Danish medical company.

**METHODS:**

The UK Medical Research Council’s guidance for process evaluation was used as a framework. Data were collected from approximately six months before the implementation until ten months after (2019–2020). A mixed method study design was used (a survey of 398 employees, a focus group of four employees and field visits on two days). Data were analyzed separately and later integrated through triangulation. We used the Fisher’s exact test in the analysis of the questionnaire.

**RESULTS:**

We assessed the implementation fidelity through four key factors: reach, dose and delivery, mechanisms of change, and context for the intervention components. Despite compliance issues, the policy component had high implementation fidelity. However, the implementation fidelity of the smoking cessation support component was low. We identified three social mechanisms influencing the employees’ responsiveness towards the policy: expectation, the social aspect of the smoking facilities, and management leadership. COVID-19 was identified as the main contextual factor affecting the implementation.

**CONCLUSIONS:**

Although not all elements of the intervention components were implemented as planned, the strict smoke-free workplace intervention is considered implemented. Further strategies can be initiated to raise implementation fidelity through better communication concerning the cessation support component, compliance, and enforcement of the policy.

## INTRODUCTION

Workplaces are considered an effective setting for preventive activities through which large groups of people can be reached^1^. Smoke-free environments including smoke-free workplaces are considered an important part of the tobacco control strategy as they protect non-smokers from exposure to smoke^2^. In addition, young employees working in smoke-free environments are less likely to begin smoking than those who are exposed to tobacco smoke at work^3,4^. Smoking policies and restrictions provide a supportive environment for people who want to quit smoking, and evidence shows that smoke-free policies in a workplace setting can reduce tobacco consumption in smokers^3,5^, leading to better productivity and fewer sick days among the employees who smoke^6,7^. Further, the implementation and enforcement of smoke-free policies can lead to equal break conditions in terms of length and frequency, and a strengthened sense of community^8,9^. In a workplace setting where many employees have less than a university level education and a higher smoking prevalence, a smoke-free policy may even have the potential to reduce the social inequalities represented by smoking^2^.

In Denmark, the Smoke-free Environment Act of 2007 permits smoking in one-person offices and indoor smoking cabins, as well as in outdoor smoking areas^10^. In recent years, several Danish municipalities and workplaces, both private and public, have implemented even stricter policies, the so-called smoke-free work workplaces and in some cases smoke-free work hours^11^. A smoke-free workplace policy does not permit smoking at the worksite, but it allows employees to leave the worksite during self-paid breaks to smoke or to use, for example, e-cigarettes or smokeless tobacco. Smoke-free work hours are applicable for all the hours the employees are supposed to work, including breaks, even if the employee is not physically at the workplace. In the Danish context, smoke-free work hours include all tobacco-related products, that is, all tobacco and nicotine products (except medicinal nicotine replacement therapy) and other related products such as e-cigarettes without nicotine. Thus, the smoke-free work hours strategy is a much stricter prevention method than other strategies and is to our knowledge an unevaluated Scandinavian phenomenon^12^.

Workplace health promotion programs (WHPPs) often experience difficulties during the implementation process, which can affect the outcome of the program^13^. As prior research has mainly focused on the effectiveness of WHPPs, little is known about how to effectively implement health promotion programs such as smoking policies in workplaces^14^.

Therefore, the purpose of this study is to evaluate the implementation of a smoke-free workplace intervention in a medical company, by examining how and to what extent the intervention components were delivered in practice.

## METHODS

### The setting and intervention

The process evaluation focuses on the implementation of a strict smoking policy intervention in a Danish medical production company with >650 employees. About 40% of the employees are blue-collar workers on the production line. The company is situated in Copenhagen, a municipality that offers free smoking cessation counseling and supports private companies in implementing smoke-free policies.

*The strict smoke-free workplace policy intervention*

From 1 January 2020, smoking and the use of e-cigarettes or heated tobacco were banned anywhere on the premises at the company’s worksite. During the daily half-hour self-paid meal break, smoking is allowed at a ‘proper distance’ from the site. In practice, this means that employees cannot smoke on sidewalks and roads that surround the site or on neighboring properties affiliated with the company. Smoking in the company’s uniforms during the self-paid break is also not permitted. Employees who want to smoke must therefore change into their own clothing. Thus, smoking during the break is challenging. The first violation of the policy results in a warning, and further violations have consequences for an employee’s employment status. The decision to ban smoking and the use of e-cigarettes or heated tobacco was made by the management.

The smoking policy is strict and lies between the definition of smoke-free work hours and the smokefree workplace policy in the sense that the policy makes it very difficult for the employees who smoke to smoke during work hours. However, because it is still technically possible to smoke during the workday, we have chosen to categorize the policy as a strict smoke-free workplace policy.

**Figure 1:**
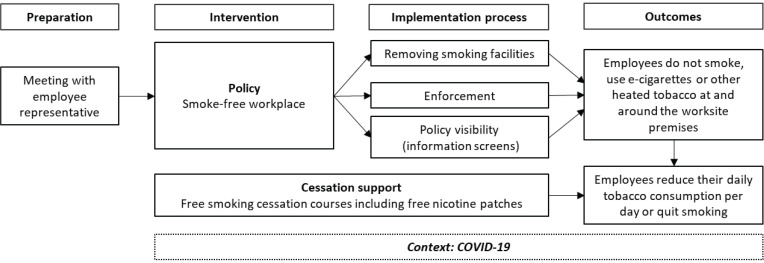
Overview of the smoke-free workplace intervention implemented in a Danish private medical company

The policy was a part of a workplace intervention that consisted of two components (Figure 1).

The policy component

This was to ensure employees do not consume cigarettes, or e-cigarettes at the company’s worksite. The policy comprised the implementation of the described smoke-free workplace policy, removal of smoking facilities (such as ashtrays, smoking sheds, and smoking area marking on asphalt), enforcement of the new policy, and internal communication about the policy.

The cessation support component

This was to help employees to better comply with the policy and/or reduce the amount of tobacco or nicotine consumption per day, or to quit completely. The component comprised smoking cessation assistance and was offered to all employees who smoked from three months before the policy was implemented (October 2019). The company offered group-based smoking cessation courses including free nicotine patches tailored to fit the shift workers. The nicotine patches were only offered for free to employees who participated in the cessation courses.

The employees were informed about the new tobacco policy 15 months (October 2018) before the implementation on 1 January 2020. The information concerning the implementation of the policy was provided through the company’s intranet and displayed on signs and information screens both internally and externally on the worksite.

### Process evaluation

We used the Medical Research Council’s (MRC) guidance for the process evaluation of complex interventions as a framework^15^. The key evaluation features regarding implementation fidelity were for this evaluation identified as: reach, dose and delivery, mechanisms of change, and context (Table 1). Implementation fidelity was conceptualized as the degree to which the policy and guidance component were implemented and delivered as intended^16^. Mechanisms of change were defined as to how social mechanisms interact and influence the implementation through the employees’ acceptance and responses^15,16^. Context included external factors which may have affected the intervention or the implementation^15,16^. The COVID-19 pandemic emerged during the implementation and thus was identified as a contextual factor for the implementation process.

**Table 1:** Key factors for implementation fidelity, and data sources used in the mixed-methods process evaluation of a smoke-free workplace intervention in a private medical company

*Factors*	*Questions*	*Indicators of fidelity*	*Data sources*
**Reach**	Were all employees who smoked reached?	**Policy** Policy visibility during implementation Knowledge about the policy among employees	Survey Focus group Observations
		**Cessation support** Knowledge about the cessation support offered	Survey Focus group
**Dose and delivery**	Were the components implemented as intended, and were the proposed criteria for implementation met?	**Policy** Smoking facilities removed No visible smoking on the worksite Employee compliance Enforcement	Survey Focus group Observations Management
		**Cessation support** Cessation courses were held Free nicotine patches	Survey Focus group Management
**Mechanisms of change**	Which social mechanisms interacted with, and influenced, the implementation?	What affected employee responsiveness?	Survey Focus group Observations
**Contextual factors**	What contextual factors interacted with, and influenced, the implementation?	How did COVID-19 affect the implementation process?	Focus group Management

### Data collection

Data were systematically collected according to the principles for process evaluation and mixed-methods research^16,17^.

*Field visits*

These were conducted before and after implementation to observe changes in the physical environment after the implementation of the smoke-free workplace policy. The first observations took place approximately six months before the implementation (20 June 2019) and the second, one month after implementation (6 February 2020). Data collection followed the principles for structured observations and informal conversations with employees^18^. The structured observations included observations on smoking visibility, observations on facilities provided for smokers, counting of cigarette butts on and around the worksite, and physical environment changes such as smoke-free workplace signs. Both field visits were made during daytime (11:00 a.m. to 4:30 p.m.). Counting of cigarette butts was done at the same time for each visit.

*Focus group interview*

This was carried out with four employees from four different divisions in October 2020, ten months after the implementation, to explore how the smoking policy influenced the employees’ everyday work-life and their perceptions of the implementation process. The interview was adapted to a video call on Teams due to the COVID-19 restrictions. The interview was performed during working hours and lasted for one hour. The facilitator organized the interview around three broad questions and encouraged interaction via open-ended questions and direct requests for participants to comment and share similar experiences.

*The electronic survey*

This was a follow-up to a baseline survey done in March 2019. The questionnaire was developed in cooperation with Copenhagen Municipality. Items related to implementation fidelity were created uniquely for this project. To ensure the appropriateness and clarity of the survey, pre-testing was done on a small number (n=7) of employees before invitations were sent. Small changes were made to the questionnaire based on the feedback. Company employees received an e-mailed invitation with a link to the follow-up questionnaire 12–13 months (January–February 2021) after the implementation of the smoke-free workplace policy.

Knowledge about the policy, engagement in the cessation support component, and information as to employees’ work situations during the COVID-19 lockdown in 2020 were collected through e-mail correspondence with the company management and are included in the assessment of the implementation process, but not in the data analysis.

*Participation*

When employees were invited to participate in the electronic survey and the focus group interview, written information about the study was given before the survey link was sent out and the interview was held. The employees were informed that participation was voluntary, that their information would be used for research purposes only and treated confidentially, and of the possibility to withdraw from the study. All participants were informed that their information would be used for publication and consented to participate.

### Data analysis

The data analysis was performed in several steps and followed the principles and procedures for triangulation in a mixed-methods study design^16,17^. The three data sets were first integrated during the interpretation of the results using the data sources as a supplement to one another.

A systematic reading of the field notes was performed, including pictures of changes in the physical environment of the worksite and notes from informal conversations with employees. The analysis of the observations was structured and coded according to the key factors for implementation fidelity shown in Table 1. This contributed to the evaluation questions regarding reach, dose, and delivery for the policy component, as well as for the mechanisms of change (Table 2).

**Table 2:** Observed key factors for implementation fidelity of a smoke-free workplace intervention in a private medical company: changes made in the workplace environment after implementation

*Indicator of fidelity*	*Observations made six months before implementation (2019)*	*Observations made one month after implementation (2020)*
**Reach**		
**Policy component**		
Policy visibility	-	Display of policy on signs and information screens both internally and externally on the worksite premises
**Dose and delivery**		
**Policy component**		
Smoking facilities removed	Three smoking areas on-site with ashtrays, benches, chairs, one shed, and marks on the asphalt pointing to the smoking areas.	Facilities partially removed: All ashtrays removed, no-smoking sign in old smoking shed. Benches and chairs (used by smokers) were not removed, and smoking signs on asphalt were not removed*.
	Many cigarette butts were observed around smoking stations (>500) and on sidewalks around the worksite (>300).	Only a few cigarette butts (<20) on-site. More cigarette butts were observed around the worksite (>200).
No visible smoking on the worksite	Smokers on-site and at every smoking station during the visit.	Few (2) visible smokers in work-clothes directly in front of the main entrance.
Employee compliance	-	Some still smoke during other breaks than the self-paid meal break.
**Mechanisms of change**		
Employee responsiveness	The policy was expected by the smokers.	-
	The smokers said that they would miss the social aspect of the smoking breaks.	-
	-	Management was perceived as ‘fair’, but employees were also careful not to say anything too critical, and asked if what they said was confidential.

* Benches and chairs (used by smokers), and smoking area marks on asphalt were removed later in the Fall of 2020.

Next, the focus group interview was transcribed verbatim. The transcription was read and coded through a thematic network analysis in multiple rounds^19^. Results from the coding process were discussed within the research group. Themes and citations related to the identified key factors for implementation fidelity were coded, while still allowing sub-themes to emerge (Supplementary file). Thereby our understanding of the extent of the fidelity of the implementation was expanded.

An analysis of the data from the electronic survey was then performed (Tables 3 and 4). This provided a deeper level of understanding of the implementation process through questions regarding reach, dose, delivery, and mechanisms of change. The responses were divided into two groups: users of tobacco or e-cigarettes (UTE) and non-users of tobacco or e-cigarettes (NUTE). Employees were categorized as UTE if they smoked conventional cigarettes or used e-cigarettes daily or at least once a week. Employees were categorized as NUTE if they never had smoked or used e-cigarettes, were ex-smokers, or smoked/used e-cigarettes less than once a week. For items related to knowledge about the intervention and the implementation (‘Do you know…?’), the answers ‘No’ and ‘I don't know’ were merged into one category ‘No’. Fisher’s exact test was used to determine differences in knowledge, attitude, perceived compliance, and perceived enforcement one year after the policy implementation. Statistically significant differences were identified at p<0.05. Statistical analyses were performed using the statistical software SAS Enterprise Guide 7.1.

**Table 3:** Characteristics of participants in the online survey evaluating the implementation fidelity of a smoke-free workplace intervention in a private medical company one year after implementation, 2021 (N=371)

*Characteristics*	*n (%)*
**Sex**	
Female	167 (45.0)
Male	204 (55.0)
**Age** (years), median (range)	45 (22–72)
**Education level**	
<12 years	40 (10.8)
Vocational education	74 (20.0)
Tertiary courses or education (2–3 years)	66 (18.0)
Tertiary education at Bachelor’s level (3–4 years)	49 (13.0)
University graduate at Master’s level or higher (>4 years)	139 (37.0)
**Employment status**	
Blue-collar employees	156 (42.0)
Duration of employment (months), median (range)	48 (1–492)
Employees with personnel responsibilities*	56 (15.1)
Employees working at the worksite during the lockdown	206 (55.5)
Employees working night shifts	49 (13.2)
**Smoking status**	
Smoker	47 (12.7)
Non-smoker	324 (87.3)

* Employees with personnel responsibilities were managers and middle managers.

**Table 4:** Progression criteria for implementation fidelity: results from the online survey one year after implementation of the smoke-free workplace intervention in a private medical company, 2021 (N=371)

*Variable*	*Response*	*Users of tobacco or e-cigarettes (N=47) n (%)*	*Non-users of tobacco or e-cigarettes (N=324) n (%)*	*Total (N=371) n (%)*	*p*
**Reach**					
**Policy component**					
Knowledge of smoking policy	Yes	33 (70.2)	226 (69.8)	259 (69.8)	0.135
	No	14 (29.8)	98 (30.3)	112 (30.2)	
Knowledge of policy for non-compliance	Yes	29 (61.7)	169 (52.2)	198 (53.4)	0.06
	No	18 (38.3)	155 (47.8)	173 (46.6)	
Satisfactory communication about policy implementation	Agree	25 (53.2)	191 (59.0)	216 (58.2)	<0.001
	Partly agree	7 (14.9)	27 (8.3)	34 (9.2)	
	Disagree	11 (23.4)	9 (2.8)	20 (5.4)	
	Don’t Know	4 (8.5)	97 (29.9)	101 (27.2)	
**Cessation support component**					
Knowledge of smoking cessation courses	Yes	30 (63.8)	205 (63.3)	235 (63.3)	0.128
	No	17 (36.2)	119 (36.7)	136 (36.7)	
Knowledge of free nicotine replacement	Yes	6 (12.8)	67 (20.7)	73 (19.7)	0.074
	No	41 (87.2)	257 (79.3)	298 (80.3)	
**Dose and delivery**					
**Policy component**					
The policy is currently a normal part of the work environment	Agree	31 (66.0)	228 (70.4)	259 (69.8)	<0.001
	Partly agree	7 (14.9)	21 (6.5)	28 (7.5)	
	Disagree	7 (14.9)	5 (1.5)	12 (3.2)	
	Don’t know	2 (4.3)	70 (21.6)	72 (19.4)	
Visibility of smoking at the worksite	Never	43 (91.5)	265 (81.8)	308 (83.0)	0.025
	Rarely	4 (8.5)	51 (15.7)	55 (14.8)	
	Often	0 (0)	8 (2.5)	8 (2.2)	
Compliance among smokers	All or most of the smokers	37 (78.7)	177 (54.6)	214 (57.7)	<0.001
	Few or none of the smokers	4 (8.5)	15 (4.6)	19 (5.1)	
	Don't know	6 (12.8)	132 (40.7)	138 (37.2)	
Smoking at the worksite	Yes	0 (0)	-	-	-
	No	43 (91.5)	-	-	
	Not answered	4 (8.5)	-	-	
Smoking at times other than the self-paid lunchbreak	Yes	11 (23.4)	-	-	-
	No	32 (68.1)	-	-	
	Not answered	4 (8.5)	-	-	
**Cessation support component**					
Satisfactory support during implementation	Yes	29 (61.7)	86 (26.5)	115 (31.0)	<0.001
	No	18 (38.3)	35 (10.8)	53 (14.3)	
	Don’t know	0 (0)	203 (62.7)	203 (54.7)	
**Mechanisms of change**					
I support the policy	Agree	21 (44.7)	230 (71.0)	251 (67.7)	<0.001
	Partly agree	6 (12.8)	33 (10.2)	39 (10.5)	
	Disagree	17 (36.2)	14 (4.3)	31 (8.4)	
	Don’t know	3 (6.4)	47 (14.5)	50 (13.5)	
My colleagues support the policy	Agree	17 (36.2)	156 (48.2)	173 (46.6)	<0.001
	Partly agree	12 (25.5)	49 (15.1)	61 (16.4)	
	Disagree	10 (21.3)	8 (2.5)	18 (4.9)	
	Don’t know	8 (17.0)	111 (34.3)	119 (32.1)	
My manager supports the policy	Agree	31 (66.0)	164 (50.6)	195 (52.6)	<0.001
	Partly agree	2 (4.3)	19 (5.9)	21 (5.7)	
	Disagree	5 (10.6)	6 (1.9)	11 (3.0)	
	Don’t know	9 (19.2)	135 (41.7)	144 (38.8)	

## RESULTS

We present the findings as a summary across data and according to the evaluation features of reach, dose and delivery, mechanisms of change, and context.

Employees in the focus group interview had been employed for between 2.5 and 10 years. One of the participants was a cigarette smoker (UTE) and three were NUTE, of which two were former smokers. All participants were blue-collar employees.

A total of 398 (59.9%) participants responded to the electronic questionnaire. Employees who did not answer items related to smoking habits and the implementation of the intervention were excluded (n=27), resulting in 371 respondents of whom 55% were men. The median age was 45 years, 42% were blue-collar employees, 55.5% of the respondents had gone physically to work as usual during the COVID-19 lockdown, 12.7% of the employees were categorized as UTE of which 8.5% (n=4) were e-cigarette users only, and 14.9% (n=7) were dual users of conventional cigarettes and e-cigarettes. The characteristics of the participants are presented in Table 3.

### Reach

*Policy component*

One month after the implementation, information about the tobacco policy of a smoke-free worksite was observed on information screens throughout the worksite, on signs near coffee machines and entrances, and on a few signs on the external worksite surroundings (Table 2).

In the focus group, ten months after the implementation, there was an overall perception that everybody knew about the policy and that the communication regarding the implementation was satisfactory.

*‘I would say that it [i.e. the communication of the policy] has been satisfactory.’* (Smoker)

After one year, more UTE than NUTE knew about the smoking policy and the policy for non-compliance, but the differences between the UTE and NUTE were not statistically significant.

Overall, most of the employees (69.8%) knew about the smoking policy one year after implementation. More UTE than NUTE knew the policy for non-compliance. There was a statistically significant difference between UTE and NUTE in their answers concerning their levels of satisfaction with the communication of the implementation. More UTE felt that the communication had in some ways failed, as a large number (23.4%) – compared to NUTE (2.8%) – disagreed that the communication had been satisfactory, whereas more NUTE answered ‘don't know’ (29.9%) (Table 4).

*Cessation support component*

Information about the cessation courses and the possibility of free nicotine patches were provided on the company’s intranet. In the focus group interview, there was confusion about whether the courses were planned to accommodate both dayshift and nightshift workers’ schedules and whether the nicotine patches were allowed in the sterile areas of the worksite.

*‘Everybody thought it was a fine idea with [i.e. to have] the cessation support groups, but the employees that work evening/nights, they were like … when should we participate? We can't do that.’* (Smoker)

*‘I’m an employee representative, so I was in the health committee and spoke to the management, and there were cessation courses planned so that everybody –no matter of the day, evening, or night –could meet. And it was multiple times.’* (Former smoker)

One year after the implementation, 63.3% of all employees knew about the smoking cessation courses, but only 19.7% knew about the opportunity of free nicotine patches (Table 4).

### Dose and delivery

*Policy component*

Within one month of the implementation, observations showed that all ashtrays had been removed from the company’s three smoking stations, but smoking facilities such as smoking areas marked on asphalt and smoking shelters had not been removed. These facilities were, however, removed during the first year of implementation. There was a dramatic reduction in the number of cigarette butts counted around the worksite one month after implementation, while there was a small reduction in the number of cigarette butts counted off the premises, immediately outside the worksite (and butts might come from other smokers, not necessarily employees) (Table 2). During the observation visit in February 2020, two employees in work clothes were spotted smoking directly in front of the main entrance. Further, we learned through informal conversations with the employees that some employees left the worksite to smoke during breaks other than the self-paid meal break, which is a violation of the policy.

Ten months after the implementation, participants in the focus group explained how warnings had been given because some employees had been smoking on the worksite, and that they knew of one employee being dismissed due to non-compliance with the tobacco policy. According to the focus group participants, an employee will first receive written warnings, and dismissal will only happen if there is repeated non-compliance by an employee.

One year after the implementation, most of the employees (69.8%) agreed that the policy had become a normal part of the work environment. However, more UTE (14.9%) than NUTE, (1.5%), disagreed that the policy had become a normal part of the work environment. Further, more NUTE (2.5%) than UTE (0%) answered that they often saw employees smoking at the worksite. More UTE (78.7%) than NUTE (54.6%) answered that all or most of the UTE complied with the policy, and more NUTE (40.7%) than UTE (12.8%) answered that they did not know. The differences in the perception of dose and delivery for the policy component between the UTE and NUTE were statistically significant, implying that the perception of the dose and delivery is associated with the use or non-use of tobacco or e-cigarettes (Table 4).

When asked about their compliance one year after the implementation, none of the UTE answered that they had been smoking or using e-cigarettes at the worksite since the policy was implemented, but 23.4% answered that they had been smoking or used e-cigarettes at other times than the self-paid meal break (Table 4).

*Cessation support component*

The group-based smoking cessation courses were planned to be held both before and after the policy implementation by counselors from Copenhagen Municipality. Only three out of six courses were held due to a lack of registrations among the employees (one course in 2019, one in January 2020, and one in January 2021). Approximately 20 employees in total participated in the cessation courses. Only five employees attending the courses used nicotine patches, even though they were offered for free.

Ten months after the implementation, three out of four focus group participants (all non-smokers) strongly agreed that tobacco or e-cigarettes users had been offered enough help to quit, even though they were not aware of anybody who had received cessation support.

More UTE than NUTE answered both yes (61.7%) and no (38.3%) to whether the support during the implementation had been satisfactory. Most NUTE (62.65%) answered that they did not know if support during the implementation had been satisfactory. The difference between UTE and NUTE was statistically significant (Table 4).

### Mechanisms of change

Through the field visits six months before and one month after the implementation, and the focus group ten months after the implementation, we identified three social mechanisms concerning the employees’ responsiveness towards the policy: expectations, the social aspect of the smoking facilities, and management leadership.

Because of the continuous societal developments regarding public health promotions in Denmark, many expected the policy before it was announced. In the focus group, it was explained, that:

*‘Every smoker has known that it would lead to this. It has been a question of time, not maybe, but when it would be smoke-free out here. There have been whispers in the corners for a while.’* (Smoker)

Further, employees mentioned that they never felt that they had a say in the policy implementation process. There was a perception that overall, it is not the employees’ job to ask questions about organizational changes; they just had to follow the rules from the management.

*‘The high-ranking management informed us that everybody thought it was a really, really good idea, and that everybody backed it up and stuff like that. I don't think that anyone would want to speak against that.’* (Former smoker)

The focus group participant explained that because many of the employees work in production, they are used to following strict rules regarding cleanliness, etc. Therefore, this was just perceived as another rule they had to follow.

Another factor in the response to and acceptance of the policy was that all the removed smoking facilities had also functioned as social gathering spots for the employees who smoked. Even though there were still facilities to sit and relax both inside and outside, meeting and socializing with colleagues from other divisions were, by the employees who smoked, perceived as more difficult and less likely to happen.

*‘I think we're missing a gathering spot because the smoking stations were where you got to know everything and got to know each other across divisions, and I really think we miss a place where you can meet.’* (Smoker)

The employees’ support and perception of their colleagues’ and managers’ support of the policy were measured as factors of mechanisms of change in the survey one year after the implementation. Although most of the employees (67.7%) agreed that they supported the policy, more NUTE (71%) than UTE (44.7%) supported the policy (Table 4). Further, more NUTE (48.2%) than UTE (36.2%) perceived that their colleagues supported the policy, whereas more UTE (66%) than NUTE (50.6%) perceived that their managers supported the policy. More NUTE did not know whether their colleagues (34.3%) or manager (41.7%) supported the policy compared to UTE (17% and 19.2%, respectively). The difference between UTE and NUTE support and perception of support from colleagues and managers was statistically significant (Table 4).

### Context

We identified COVID-19 as the only contextual factor implicating the implementation. Not long after the implementation of the new smoking policy, the COVID-19 pandemic resulted in two national lockdown periods. During the lockdown periods about 12% of the employees, mostly white-collar employees, worked from home. The company’s production was unaffected by the lockdowns. That means that 88% of the employees (mostly blue-collars) went to work as usual and therefore had to follow the smoke-free worksite policy. At the worksite, employees were divided into break groups to be able to socially distance themselves.

## DISCUSSION

This mixed-methods study examined the implementation fidelity of a smoke-free workplace intervention, using as a case study the example of a Danish medical company.

Taking an overall look at the implementation fidelity of the two intervention components, we found that implementation fidelity was quite high for the reach of the policy component. Almost all employees knew about the strict smoke-free workplace policy, and more than half of all employees supported it. Many UTEs complied with the policy, but there were also observed violations of the policy. There were several statistically significant differences between the UTE and NUTE as to levels of support and perceptions of the implementation process. These differences correlate with a recent study in a university setting which found lower support for a smoke-free university policy among tobacco users, and non-users had less knowledge about the policy, compliance, and enforcement than tobacco users^20^.

During the first year of implementation, all outdoor smoking facilities and signs were completely removed. Despite the high level of knowledge of the policy and the perception that the policy one year after its implementation was considered a normal part of the work environment, not all complied with the policy. Some employees were still smoking or used e-cigarettes at the worksite and during breaks other than the self-paid meal break, suggesting only mediocre implementation fidelity for the dose and delivery factor of the policy component.

The implementation fidelity regarding the cessation support component was low. Only three out of six planned cessation courses were held from 2019 to 2021. The confusion concerning both the cessation courses and the free nicotine patches suggests that the reach to smokers was insufficient, resulting in low implementation fidelity concerning dose and delivery. Thus, the intended impact mechanism to prompt smokers to quit smoking instead of just leaving the worksite during meal breaks was not implemented. Kava et al.^21^ describe difficulties with persuading smokers to participate in cessation activities offered in smaller companies. According to a systematic review by Wierenga et al.^13^ of process evaluations of health promotion programs, a reason for an insufficient reach of participants can be a lack of communication about the program, which can lead to low participation levels.

The expectation of the policy implementation^22^, and the notice more than a year in advance, prepared smokers for a strict non-smoking policy. Our findings correlate with previous studies in a psychiatric setting, where adequate planning and preparation time were associated with the greater success of smoke-free policies^23^.

### Barriers to implementation

The employees’ lack of knowledge as to how the policy would be enforced, as well as – among the employees who smoked – lower levels of support and adherence to the policy, may have been a barrier to the implementation^20,21,23^.

Despite the introduction of the strict smoke-free workplace policy, the employees who smoke still, to date, have the opportunity of smoking during the workday. If the policy instead completely prohibited smoking during work hours, employees would not have had the opportunity to smoke during their workday, and the policy would be easier to enforce, as there would be no ‘gray areas’ for when or where smoking is allowed. To date, it remains an ongoing temptation for smokers to leave the workplace to smoke, and this undermines their smoking cessation intentions. Further, due to the obligatory change of clothes, smokers have to decide either to smoke or to have a meal break. A review on barriers to the introduction of smoke-free workplaces in Central Europe concludes that when implementing a smoke-free policy, ‘no exceptions should be made, as they serve as a barrier to a smoke-free working environment’^24^. However, smoke-free work hours are difficult to implement in the private sector where employees have self-paid breaks, and a company cannot control what their employees do in their free time.

To our knowledge, the company did not purposefully use any other strategies to form or improve a supportive environment amongst employees regarding the policy and nicotine cessation support components. A more effective way might have been, before implementation, to focus on creating a greater sense of understanding of the components and the importance of the policy in the work environment – for instance, in meetings where all aspects of the policy could have been discussed or by posters describing the positive aspects of the policy and leaflets placed in the outdoor smoking areas, describing the smoking cessation support offered. Studies show that if the communication concerning the cessation support component had been more visible, or if there had been a mandatory introduction for employees to the cessation support component, the perception of support may have been higher as well as the rate of participation in the cessation courses^1^.

During our field visits and in the focus group, the smoking employees said that they were missing the social aspect of the smoking breaks. Studies suggest that smoking policies in a workplace setting can lead to more equal break conditions and a strengthened sense of community across all employees, both non-smoking and those who smoked^8,9^. Social activities to substitute the social aspect of the smoking breaks may have improved the smokers’ perception of community at the workplace, but these were not initiated due to the COVID-19 restrictions and strict guidelines for social distancing.

There is strong evidence showing that consistent and visible supportive involvement from management has a great (and positive) impact on the implementation of a workplace health policy and on employee acceptance and responsiveness^23,25-27^. Instead of feeling involved in the decision-making and implementation process, the employees in our study accepted the policy because they felt they had to, and not because they all necessarily supported it. It is noteworthy that a higher percentage of UTE than NUTE reported that their (middle) managers did not support the policy. We have not examined if this was true or just a perception.

According to the MRC, understanding context interdependence in a given intervention is critical to the success or failure of implementation^28^. Since all the production (involving 88% of employees) was not affected by the lockdown periods, COVID-19 may, contrary to our expectations, not have had a big impact on the implementation of the policy component.

### Strengths and limitations

Four out of ten employees did not complete the electronic survey and a selection bias must be expected, probably involving the less educated employees and smokers who disagree with the policy. Misinformation is a potential problem in all surveys, but as the survey was anonymous and performed by researchers, not the management, we hope that levels of misinformation were low. The employees were informed that answers were confidential, and no information would be given to the employer. Few smokers were included which made it difficult to stratify, for example by sex, age, department, or worker/manager.

Furthermore, although the process evaluation was planned ahead of the implementation, which is a strength^13^, the evaluation ended up being less systematic than intended. Due to accommodating social distancing rules and lockdown during the COVID-19 pandemic, it was not possible to visit the company as many times as planned; and, in particular, the structured observations regarding the change in the physical environment were not performed as intended. We planned to recruit participants to the focus group interview during the field visits, but this was difficult since many of the observational visits were cancelled. Further, recruitment to the online focus group was also difficult since not all employees working in production had access to a computer through their employment. A more diverse focus group with more than one UTE, would have been optimal.

## CONCLUSIONS

Although not all key factors were implemented as intended, the strict smoke-free workplace policy was implemented with high fidelity and resulted in changes in the work environment at the company’s worksite. Based on this study, which focused on the implementation process, we suggest that companies that want to implement a smoke-free workplace policy focus on involving the employees in the communication and planning of the policy implementation before the actual implementation. Hereby it is possible to explore what can be done to improve participant response to the policy, and possible activities included, such as cessation support courses. Further, we suggest considering strategies to involve the social aspect of smoking breaks and explore how the employees who are smokers can continue to feel a sense of community without having to smoke.

## Data Availability

The datasets used and/or analyzed during the current study are available from the corresponding author on reasonable request.
