# Corrigendum: Implementation fidelity of a smoke-free workplace intervention in a private medical company: A mixed-methods process evaluation

**DOI:** 10.18332/tpc/173032

**Published:** 2023-10-04

**Authors:** Sofie K. B. Rasmussen, Lærke P. Lidegaard, Charlotta Pisinger, Nina F. Johnsen, Maria Kristiansen

**Affiliations:** 1 Center for Clinical Research and Prevention, Bispebjerg-Frederiksberg University Hospital, Capital Region of Denmark, Frederiksberg, Denmark; 2 Department of Research, Danish Heart Foundation, Copenhagen, Denmark; 3 Department of Public Health, Faculty of Health and Medical Sciences, University of Copenhagen, Copenhagen, Denmark; 4 Department of Public Health and Center for Healthy Aging, Faculty of Health and Medical Sciences, University of Copenhagen, Copenhagen, Denmark

**Keywords:** smoke-free policies, smoke-free workplace, smoke-free work hours, tobacco-free workplace, implementation research, mixed methods study

Corrigendum on:

Implementation fidelity of a smoke-free workplace intervention in a private medical company: A mixed-methods process evaluation

By Sofie K. B. Rasmussen, Lærke P. Lidegaard, Charlotta Pisinger, Nina F. Johnsen, Maria Kristiansen

Tobacco Prevention and Cessation, Volume 9, Issue May, Pages 1-13, Published date: 26 May 2023

DOI: https://doi.org/10.18332/tpc/162878

In the originally published version of the article, the name of the second author has changed to Lærke P. Lidegaard. The mentioned changes have been corrected also online.

