# Supplementary material for: Implementation fidelity of a smoke-free workplace intervention in a private medical company: A mixed-methods process evaluation
Source: Tob Prev Cessat. 2023 May 26;9:17. doi: 10.18332/tpc/162878 (PMC10214475; doi:10.18332/tpc/162878)
Supplement: Supplementary file 1 [file TPC-9-17-s1.pdf]

Additional file 1

Presented are the results from the thematic network analysis of the focus group interview.

Key factors for implementation fidelity, focus group

Factor/indicator	Data condensation	Quotes
**Reach**		
Policy component		
Information about the policy from direction to management to employee	The perception is that every employee knows about the policy and is aware of the rules for enforcement.	*"Yes, something happens if you break the policy"* (Non-smoker).
	The agreement that the communication about the policy has been satisfying.	*" I would say that it [i.e. the communication of the policy] has been satisfactory" (Smoker).*
Cessation support component		
Information about cessation support	There is a misconception about the cessation support groups and when they are being held	*"Everybody thought it was a fine idea with [i.e. to have] the cessation support groups, but the employees that work evening/nights, they were like… when should we participate? We can't do that"* (Smoker).<br><br>*"I'm an employee representative, so I was in the health committee and spoke to the management, and there were cessation courses planned so that everybody—no matter of the day, evening, or night—could meet. And it was multiple times"* (Former smoker).
	There is confusion as to if nicotine patches are allowed in the sterile areas	*"I don't know whether it has been an issue for people, I just know that it was brought up in relation to the filling operators, and in some cases it could have been smart to make some sort of plan for it, right?"* (Smoker).
**Dose & delivery**		
Policy component		
No visible smoking on the worksite	Smokers are now changing clothes and standing on the other side of the street when they smoke during their break(s)	*"We are three that smoke in the lunch break, so since March we have been out from the* [work]*site and eaten our lunch out in the open, and then we have smoked our cigarettes. Out two cigarettes we have time for while we eat our lunch and then we go back again"* (Smoker).

Enforcement	There have been given warnings and been one suspension for those that do not comply with the policy	*"There is at least one expulsion and then there are some written warnings"* (Smoker).<br><br>*"Those that have been given a written warning, they have been told several times, 'hey you must go out* [of the worksite*] to smoke' or it has been because they have gone out to smoke in their work clothes* […] *They definitely have not been expelled the first time it happened, it's because more violations have happened. The expulsion that I know of, is someone who has smoked on the site several times, you know?"* (Smoker).
	The rules apply to all employees, managers, and directors	*"If our director stands in the middle of the yard and smokes, she will also be kicked out"* (Former smoker).
Cessation support component		
Cessation courses and free nicotine patches	The perception is that everybody has been offered the help they could have needed, even though the response to cessation support has been low.	*"Everybody should get as much help they want, but then I think damn it is hard to do more* […]*, other than offer alternatives to smokers, offer smoking cessation courses, offer people that they get off with pay to take the cessation courses. Plan them as often as they possibly could be, there was no limit for one cessation course"* (ex-smoker)<br><br>*"I don't know if the help should have been different for someone that smokes 60 cigarettes a day, and for someone that smokes – I don't know – 10 cigarettes a day".* (Smoker).
**Mechanisms of change**		
Employee responsiveness		
Expectancy	The implementation was expected and for the most, they understand and accept the policy	*"Every smoker has known that it would lead to this. It has been a question of time, not maybe, but when it would be smoke-free out here. There have been whispers in the corners for a while"* (Smoker).
Management/leadership	A perception that management has the final say. Responsiveness, not support, is highly affected by this.	*"The high-ranking management informed us that everybody thought it was a really, really good idea, and that everybody backed it up and stuff like that. I don't think that anyone would want to speak against that"* (Former smoker).<br><br>*"We're an organization where not everything makes sense, so... [...] it's not allowed for us to*

		start questioning strange rules, but it's uhm… because there usually is a meaning to the madness, right? So therefore, there's perhaps greater confidence that when some guidelines are put out there, well then that's what we will do, we don't question it to the same extent as you'd do in some industrial warehouse, or a store, or whatever…" (Former smoker).
The social aspect of the smoking facilities	When they moved the smoking stations, they removed a social spot. There is now a perception among smokers, that socializing between departments is more difficult now.	*"I think we're missing a gathering spot because the smoking stations were where you got to know everything and got to know each other across divisions, and I really think we miss a place where you can meet"* (Smoker).<br><br>*"Now you don't have an apology anymore, so you will not get out, there is no place. It often rains, there is no place to be then"* (Smoker).
**Contextual factors**		
COVID-19	Have influenced the social aspect, but the implementation has been going on for longer than the pandemic.	*"Well, of course, I think Covid has done its part so that you can't [socialize]"* (Smoker).<br><br>*"It is of course contributing, but now we have been smoke-free for somewhat longer than the pandemic, right?"* (Former smoker).

Standards for Reporting Implementation Studies: the StaRI checklist for completion

The StaRI standard should be referenced as: Pinnock H, Barwick M, Carpenter C, Eldridge S, Grandes G, Griffiths CJ, Rycroft-Malone J, Meissner P, Murray E, Patel A, Sheikh A, Taylor SJC for the StaRI Group. Standards for Reporting Implementation Studies (StaRI) statement. *BMJ* 2017;356:i6795

The detailed Explanation and Elaboration document, which provides the rationale and exemplar text for all these items is: Pinnock H, Barwick M, Carpenter C, Eldridge S, Grandes G, Griffiths C, Rycroft-Malone J, Meissner P, Murray E, Patel A, Sheikh A, Taylor S, for the StaRI group. Standards for Reporting Implementation Studies (StaRI). Explanation and Elaboration document. *BMJ Open* 2017 2017;7:e013318

Notes: A key concept of the StaRI standards is the dual strands of describing, on the one hand, the implementation strategy and, on the other, the clinical, healthcare, or public health intervention that is being implemented. These strands are represented as two columns in the checklist.

The primary focus of implementation science is the implementation strategy (column 1) and the expectation is that this will always be completed.	The evidence about the impact of the intervention on the targeted population should always be considered (column 2) and either health outcomes reported or robust evidence cited to support a known beneficial effect of the intervention on the health of individuals or populations.

The StaRI standardsrefers to the broad range of study designs employed in implementation science. Authors should refer to other reporting standards for advice on reporting specific methodological features. Conversely, whilst all items are worthy of consideration, not all items will be applicable to, or feasible within every study.

Checklist item		Reported (X)	Implementation Strategy	Reported (X)	Intervention
			"Implementation strategy" refers to how the intervention was implemented		"Intervention" refers to the healthcare or public health intervention that is being implemented.
**Title and abstract**					
Title	**1**	**X**	Identification as an implementation study, and description of the methodology in the title and/or keywords		
Abstract	**2**	**X**	Identification as an implementation study, including a description of the implementation strategy to be tested, the evidence-based intervention being implemented, and defining the key implementation and health outcomes.		
**Introduction**					
Introduction	**3**	**X**	Description of the problem, challenge or deficiency in healthcare or public health that the intervention being implemented aims to address.		
Rationale	**4**	**X**	The scientific background and rationale for the implementation strategy (including any underpinning	**X**	The scientific background and rationale for the intervention being implemented (including evidence

			theory/framework/model, how it is expected to achieve its effects and any pilot work).		about its effectiveness and how it is expected to achieve its effects).
Aims and objectives	**5**	**X**	The aims of the study, differentiating between implementation objectives and any intervention objectives.		
**Methods: description**					
Design	**6**	**X**	The design and key features of the evaluation, (cross referencing to any appropriate methodology reporting standards) and any changes to study protocol, with reasons		
Context	**7**	**X**	The context in which the intervention was implemented. (Consider social, economic, policy, healthcare, organisational barriers and facilitators that might influence implementation elsewhere).		
Targeted 'sites'	**8**	**X**	The characteristics of the targeted 'site(s)' (e.g locations/personnel/resources etc.) for implementation and any eligibility criteria.	**X**	The population targeted by the intervention and any eligibility criteria.
Description	**9**	**X**	A description of the implementation strategy	**X**	A description of the intervention
Sub-groups	**10**	Not applicable	Any sub-groups recruited for additional research tasks, and/or nested studies are described		
**Methods: evaluation**					
Outcomes	**11**	**X**	Defined pre-specified primary and other outcome(s) of the implementation strategy, and how they were assessed. Document any pre-determined targets	**X**	Defined pre-specified primary and other outcome(s) of the intervention (if assessed), and how they were assessed. Document any pre-determined targets
Process evaluation	**12**	**X**	Process evaluation objectives and outcomes related to the mechanism by which the strategy is expected to work		
Economic evaluation	**13**	Not applicable	Methods for resource use, costs, economic outcomes and analysis for the implementation strategy	Not applicable	Methods for resource use, costs, economic outcomes and analysis for the intervention
Sample size	**14**	Not applicable	Rationale for sample sizes (including sample size calculations, budgetary constraints, practical considerations, data saturation, as appropriate)		
Analysis	**15**	**X**	Methods of analysis (with reasons for that choice)		

Sub-group analyses	**16**	Not applicable	Any a priori sub-group analyses (e.g. between different sites in a multicentre study, different clinical or demographic populations), and sub-groups recruited to specific nested research tasks		
**Results**					
Characteristics	**17**	**X**	Proportion recruited and characteristics of the recipient population for the implementation strategy	**X**	Proportion recruited and characteristics (if appropriate) of the recipient population for the intervention
Outcomes	**18**	**X**	Primary and other outcome(s) of the implementation strategy	Not applicable	Primary and other outcome(s) of the Intervention (if assessed)
Process outcomes	**19**	**X**	Process data related to the implementation strategy mapped to the mechanism by which the strategy is expected to work		
Economic evaluation	**20**	Not applicable	Resource use, costs, economic outcomes and analysis for the implementation strategy	Not applicable	Resource use, costs, economic outcomes and analysis for the intervention
Sub-group analyses	**21**	Not applicable	Representativeness and outcomes of subgroups including those recruited to specific research tasks		
Fidelity/ adaptation	**22**	**X**	Fidelity to implementation strategy as planned and adaptation to suit context and preferences	**X**	Fidelity to delivering the core components of intervention (where measured)
Contextual changes	**23**	**X**	Contextual changes (if any) which may have affected outcomes		
Harms	**24**	Not applicable	All important harms or unintended effects in each group		
**Discussion**					
Structured discussion	**25**	**X**	Summary of findings, strengths and limitations, comparisons with other studies, conclusions and implications		
Implications	**26**	Not applicable	Discussion of policy, practice and/or research implications of the implementation strategy (specifically including scalability)	**X**	Discussion of policy, practice and/or research implications of the intervention (specifically including sustainability)
**General**					
Statements	**27**	**X**	Include statement(s) on regulatory approvals (including, as appropriate, ethical approval, confidential use of routine data, governance approval), trial/study registration (availability of protocol), funding and conflicts of interest		

© 2023 Rasmussen S. K. B. et al.
